# High Yielding Continuous-Flow Synthesis of Norketamine

**DOI:** 10.1021/acs.oprd.1c00407

**Published:** 2022-03-26

**Authors:** Marcos Veguillas Hernando, Jonathan C. Moore, Rowena A. Howie, Richard A. Castledine, Samuel L. Bourne, Gareth N. Jenkins, Peter Licence, Martyn Poliakoff, Michael W. George

**Affiliations:** † School of Chemistry, University of Nottingham, University Park, Nottingham NG7 2RD, U.K.; ‡ Quotient Sciences, Taylor Drive, Alnwick, Northumberland NE66 2DH, U.K.; § GSK Carbon Neutral Laboratories for Sustainable Chemistry, University of Nottingham, Nottingham NG7 2GA, U.K.

**Keywords:** norketamine, continuous manufacturing, bromination and amination

## Abstract

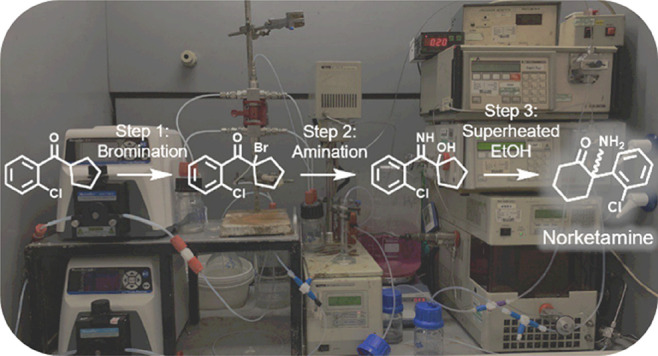

A new continuous-flow process is presented for synthesis of the pharmaceutical intermediate norketamine (**5**). Our approach has been to take the well-established and industrially applied batch synthetic route to this promising antidepressant precursor and convert it to a telescoped multi-stage continuous-flow platform. This involves the α-bromination of a ketone, an imination/rearrangement sequence with liquid ammonia, and a thermally induced α-iminol rearrangement. Our approach is high yielding and provides several processing advantages including the reduction of many of the hazards conventionally associated with this route, particularly in the handling of liquid bromine, hydrogen bromide gas, and liquid ammonia. Each of these presents serious operational challenges in a batch process at scale.

## Introduction

Continuous processing is becoming increasingly attractive for the manufacture of medicines because it offers opportunities for faster process development and safer handling of hazardous reagents.^1^ Over the past two decades, continuous-flow chemistry has become commonplace in both academia and industry and nowadays it pervades the whole process of research, from reaction discovery and optimization to scale-up and production of fine chemicals and active pharmaceutical ingredients.^2^ The increasing interest in this field stems from the many advantages that synthesis in-flow can offer. Specifically, processes can often be made safer, greener, and more efficient when performed in continuous flow. These benefits are particularly apparent when working with hazardous materials, where the scalability of a process in a batch format can be problematic.^3,4^ Flow chemistry also offers flexibility in reconfiguring reactors to adapt to changing manufacturing requirements. This is particularly useful in the context of personalized medicine, which leads to the production of a larger number of compounds but in relatively small amounts. In this paper, we demonstrate how flow chemistry can simplify the synthesis of norketamine (**5**), a pharmaceutically relevant metabolite of the antidepressant, ketamine (**4**). We take advantage of flow chemistry to minimize the risks of handling toxic reagents such as ammonia and molecular bromine on a kilo scale.

In recent years, ketamine (**4**) and its derivatives have been identified as a revolutionary new class of antidepressants. Depression is a hugely important and poorly understood condition that affects over 100 million people.^5^ It is also the most common psychiatric condition in people who commit suicide;^6^ which is itself one of the leading causes of death worldwide.^7^ Many of the most common medications for depression, such as selective serotonin reuptake inhibitors, will typically take weeks of constant exposure before they become effective. This is exacerbated by the fact that around one-third of people do not respond to two or more antidepressant therapies (treatment-resistant depression).^8^ Unlike selective serotonin reuptake inhibitors, ketamine (**4**) has been shown to have extremely fast acting effects^9−12^ and reduce multiple measures of suicidality in patients with treatment-resistant depression.^13−17^ Ketamine (**4**) and its derivatives are characterized by their arylcyclohexylamine structure and are antagonists of the *N*-methyl-d-aspartate receptor. Norketamine (**5**) is a major metabolite of ketamine (**4**) that is produced by cytochrome P450 enzymes in the liver. It goes on to be hydroxylated to form (2*R*,6*R*)-hydroxynorketamine. The latter compound also has a promising antidepressant activity but differs from the aforementioned derivatives in the sense that it is inactive both as an anesthetic and a psychostimulant.^11^ These are of course highly favorable properties and (2*R*,6*R*)-hydroxynorketamine is currently under development by the National Institute of Mental Health. As of late 2019, this compound is in phase I clinical trials.^18^

**Scheme 1:**
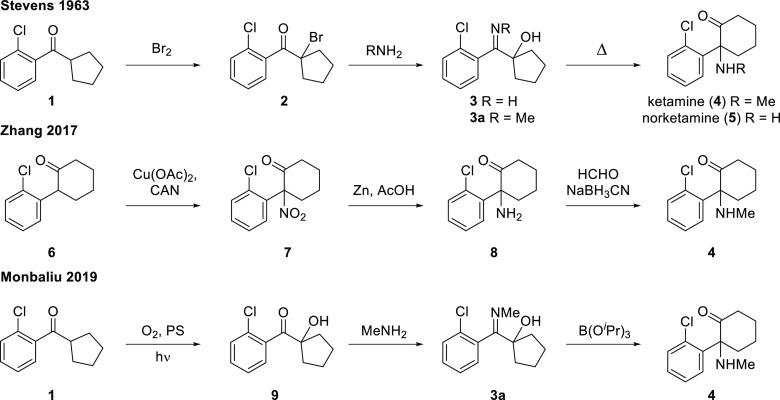
Previous Routes to Ketamine (**4**) and Norketamine (**5**)

Norketamine (**5**) is a key synthetic precursor to (2*R*,6*R*)-hydroxynorketamine and many other derivatives and is itself synthesized by the seminal route developed by Stevens in the early 1960s^19−25^ (Scheme 1). This sequence of bromination/amination/α-iminol rearrangement has remained the chosen approach for access to the arylcyclohexylamine scaffold for almost 70 years. With the increasing interest in these privileged derivatives, several methods have been reported more recently. In 2017, Zhang and co-workers reported a copper-assisted direct nitration of cyclic ketones with CAN, which enabled a short synthesis of ketamine (**4**) and norketamine (**5**) (Scheme 1).^26^ In 2019, Monbaliu and co-workers^27^ reported an innovative approach using a continuous-flow photochemical step to install the hydroxy group via α-hydroxylation of ketone **1** to provide a precursor for the amination process and subsequent ring expansion to yield ketamine (**4**).

The seminal route to norketamine (**5**) described by Stevens (Scheme 1) involves bromine and ammonia, each highly toxic and corrosive reagents with considerable material compatibility issues. Somewhat surprisingly, continuous-flow variants of this process have remained significantly underexplored. Herein, we report the development of a partially daisy-chained, multi-reactor approach for the synthesis of norketamine (**5**) combining the robust and well-established methodology reported by Stevens and continuous-flow chemistry. It should be noted that only a handful of continuous-flow reactions with bromine have been described in the literature, and to our knowledge, this is the first example of the use of liquid ammonia in continuous-flow chemistry.

### Bromination

One challenge associated with continuous-flow bromination reactions is the highly toxic and corrosive nature of Br_2_. This species corrodes steel and is therefore incompatible with much conventional flow-chemistry apparatus such as steel pipes and pumps with metallic wetted parts. Tantalum is resistant to Br_2_ but the expense of that approach can be prohibitive. In the α-bromination of ketones, an added challenge is the production of hydrogen bromide gas, which is also highly corrosive to steel and must be scrubbed on the exit of the reactor. In 2012, a continuous-flow procedure for the α-bromination of acetophenone was reported, but, this approach made use of a microreactor which would be unsuitable for the scales that we were targeting.^28^ Due to the aforementioned challenges, it was decided not to use a tubular reactor design but to instead employ a continuously stirred tank reactor (CSTR). An important consideration in CSTR reactor design is that the ratio of starting material to product in the exit stream of the reactor is largely determined by three factors: reactor volume, the flow rate, and the reaction rate. For this reason, the dimensions and productivity of the system are highly dependent on the rate of the reaction. The slower the reaction, the larger the vessel and/or the slower the flow rate required to achieve the same conversion to the product. In the case of the α-bromination of ketones with Br_2_, the reaction is catalyzed by HBr which as mentioned is itself a by-product of the reaction. Consequently, the process is autocatalytic and d[product]/dt is directly proportional to [HBr].^28^ This leads to the sigmoid reactant-concentration curves typically observed in autocatalysis and the reaction experiences a long induction period. Importantly, however, the induction period can be overcome by charging the reaction vessel with a catalytic quantity of HBr prior to the reaction. In this case, the reaction proceeds at a very high rate and the process is, therefore well suited for a CSTR reactor design.

**Figure 1:**
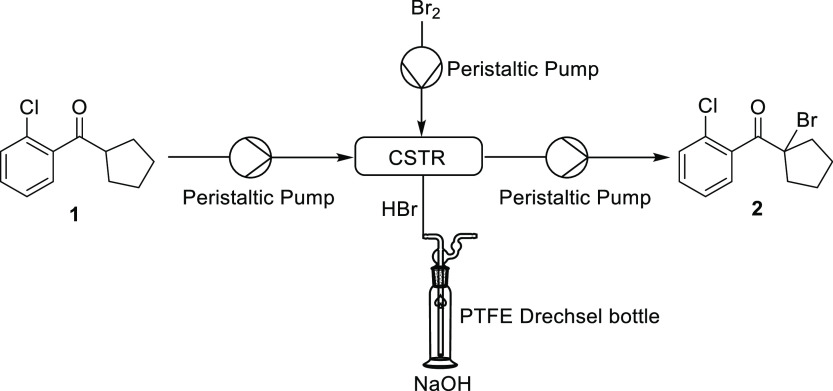
Bromination of **1** with Br_2_ in a CSTR to form the α-bromoketone **2**. 4 mm OD PTFE tubing was connected via PTFE GL14 screw threads to a round-bottom flask (which served as the reactor) with GL14/B19 quick-fit adaptors.

We envisaged that a conventional four-necked round-bottomed flask could be employed as a CSTR. Due to the corrosive nature of the reaction mixture, peristaltic pumps with PTFE tubing were selected, as this design has no metallic wetted parts. One pump was used to deliver the substrate solution, a second for the bromine solution and a third for removing the reaction mixture from the vessel (Figure 1). PTFE tubing, which is resistant to bromine and HBr, was connected to the vessel using standard GL14/B19 quick-fit adaptors. The fourth neck was connected to a PTFE Drechsel bottle containing concentrated aqueous sodium hydroxide solution to quench the hydrogen bromide gas.

To maintain a constant volume of reaction mixture in the reactor, matched flow rates were used for each of the inlet pumps and the outlet pump was set to twice this value. To vary the stoichiometry, the relative concentrations of the feed solutions in CH_2_Cl_2_ were adjusted. Accordingly, to achieve 1.1 equiv of bromine, a 4.9 M solution of bromine and a 4.5 M solution of the starting material **1** were employed (Table 1, Entry 1). When these conditions were investigated with a 10 mL reaction volume, at 0.96 mL/min total flow rate, several issues were observed. Initially, due to the high concentration and low reaction volume, considerable effervescence was observed due to the release of HBr gas. This in turn led to gas bubbles in the exit stream from the reactor, which affected the true flow rate of the reaction mixture. To address this issue, the reaction was attempted at a reduced concentration. A 1.5 M solution of **1** and a 1.6 M bromine solution were employed, which again equates to 1.1 equiv of bromine (Table 1, Entry 2). In order to maintain a comparable residence time and productivity, as the concentration was reduced by a factor of 3 and the reaction volume and flow rate were each increased by a factor of 3 (Table 1, Entry 2). Under these conditions the effervescence was reduced significantly and the reaction mixture could be efficiently pumped out of the reactor without gas bubbles in the liquid stream. Under these conditions, however, a small quantity of the starting material could be detected by thin-layer chromatography performed on a sample from the exit stream of the reactor. To address this, the reaction was repeated with a slightly more concentrated solution of bromine, 1.8 M as opposed to 1.6 M, which provided 1.2 equivalents of bromine. Under these conditions, the reaction proceeded to full conversion and following aqueous work-up with sodium hydroxide solution, the targeted bromide **2** was obtained in a quantitative isolated yield with no need for further purification. This equates to 0.89 kg/day of theoretical productivity.

**Table 1:** Results for the Continuous-Flow Bromination of **1**

entry	reaction volume (mL)	substrate flow rate (mL/min)	substrate conc. (M)	Br_2_ conc. (M)	Br_2_ equivs	conversion (%)	isolated yield (%)
1	10	0.48	4.5	4.9	1.1		
2	30	1.44	1.5	1.6	1.1		
3	30	1.44	1.5	1.8	1.2	>95%	quant.

### Amination

As in the case of bromine, the handling and use of liquid ammonia in continuous processes can be challenging, mainly due to its highly toxic and corrosive nature. Regarding safety, all the fittings used in the system were tested using a pH indicator and as an additional control measure, the reactor was placed within a water-filled reservoir containing phenolphthalein as a pH indicator. To avoid the energy intensive requirement of cooling the reaction to keep the ammonia in the liquid phase, the process was instead operated at 30 bar pressure at room temperature. Accordingly, high pressure equipment and protocols were required throughout this stage, with alternative approaches using aqueous ammonia producing low yields.

**Figure 2:**
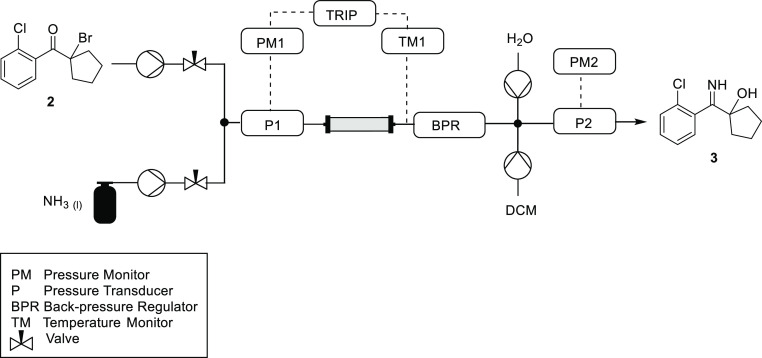
Reactor scheme for the continuous-flow imination/rearrangement sequence with liquid ammonia for conversion of the α-bromoketone **2** to the α-hydroxy imine **3**. The substrate solution and ammonia were pumped with a Jasco (PU-980) high-performance liquid chromatography pump and a chilled Jasco pump (PU-1580-CO_2_), respectively. These liquid streams were connected to the reactor via 1/16″ stainless-steel tubing and a Swagelok union.

The substrate solution was pumped into a T-piece, where it was combined with a stream of liquid ammonia (Figure 2). The combined reagents were then delivered into the reactor, which consisted of a piece of 1/4″ stainless-steel tubing. The reactor was maintained at ambient temperature by submersion in a water bath. It was determined in preliminary experiments that performing the reaction at elevated temperatures resulted in decomposition of the product. Downstream of the reactor was placed a temperature monitor and a back-pressure regulator (BPR) that was attached to the reactor using 1/16″ tubing. The pressure was maintained at 30 bar, which ensured that the ammonia was in the liquid phase. In preliminary experiments, we observed blockages in the system, which were attributed to the precipitation of the by-product ammonium bromide upon evaporation of the ammonia at the BPR. To combat this, the design was modified to include two flushing pumps that were connected to the crude stream after the BPR by means of a crosspiece (Figure 2). The dichloromethane pump ensured that the product remained soluble, whereas the water pump ensured solubility of the ammonium bromide.

Using this setup, we began screening conditions with a 0.2 M solution of the substrate in CH_2_Cl_2_ which was pumped at 0.1 mL/min, with a 0.5 mL/min flow rate of ammonia (Table 2, Entry 1). Under these conditions, the imine **3** could be observed in 89% yield alongside remaining starting material, as determined by ^1^H NMR analysis with an internal standard. When the reaction was repeated in the absence of a standard, an isolated yield of 82% could be achieved under the same conditions by precipitation of the product from a mixture of CH_2_Cl_2_ and heptane (Table 2, Entry 1). Although this was a satisfactory preliminary result, these conditions equated to 890 equiv of ammonia and the theoretical productivity was modest at 6 g/day. In an attempt to reduce the number of equivalents of ammonia, the effect of a reduction in the ammonia flow rate was investigated. It should be noted that this leads to increased residence time. Reductions in the ammonia flow rate from 0.5 to 0.2 mL/min had a negligible effect on the yield (Table 2, Entries 2–4). A further reduction in the ammonia flow rate to 0.1 mL/min, however, led to significantly reduced NMR conversion and yield (Table 2, Entry 5). Accordingly, the optimal ammonia flow rate for this experimental setup was determined to be 0.3 mL/min, which equates to a reduction to 356 equiv of ammonia (Table 2, Entry 4).

**Table 2:** Results for the Continuous-Flow Imination/Rearrangement Sequence for Conversion of the α-bromoketone **2** to the α-Hydroxy Imine **3**

entry	substrate flow rate (mL/min)	substrate conc. (M)	equivs of ammonia	flow rate ammonia (mL/min)	residence time^a^ (min)	maximum theoretical productivity^b^ (g/day)	conversion^c^ (%)	yield (%)^c^
1	0.1	0.2	890	0.5	9.28	6	93	89 (82)^d^
2	0.1	0.2	712	0.4	11.14	6	90	83
3	0.1	0.2	532	0.3	13.93	6	>95	92
4	0.1	0.2	355	0.2	18.56	6	93	92
5	0.1	0.2	178	0.1	27.85	6	56	55
6	0.1	0.6	178	0.3	13.93	19	>95	>95
7	0.1	1.2	89	0.3	13.93	39	>95	>95
8	0.1	2.4	45	0.3	13.93	77	>95	>95
9	0.2	2.4	23	0.3	11.14	155	94	90
10	0.3	2.4	15	0.3	9.28	232	64	61

a Residence times were estimated from the flow rates and densities of the reactants.

b Maximum theoretical productivity was calculated using the concentration and flow rate of the substrate solution, assuming quantitative conversion to the product.

c Conversion and yield were determined by ^1^H NMR using 1,3,5-trimethoxybenzene as an internal standard.

d Isolated yield.

Next, attempts were made to further reduce the equivalents of ammonia and also improve the productivity, namely, by increasing the substrate concentration. It should be noted that in previous experiments, before the addition of the second flushing pump, this had resulted in severe blockages. At 0.3 mL/min ammonia, the substrate concentration could be increased from 0.2 to 2.4 M, without any sign of blockages or precipitated material (Entries 6–8). Crucially, full conversion to the desired product was observed in each of these experiments.

Finally, an attempt was made to further reduce the equivalents of ammonia and increase the productivity by increasing the substrate flow rate. Increasing the flow rate to 0.2 and 0.3 mL/min led to a drop in the conversion, and the starting bromide **2** could be observed in the reaction mixture (Entries 9–10). Accordingly, the optimal conditions were deemed to be 2.4 M substrate at 0.1 mL/min flow rate with 0.3 mL/min ammonia flow rate (Entry 8). Under these optimized conditions, full conversion to the desired imine was observed with 45 equiv of ammonia at maximum theoretical productivity of 77 g/day.

### Daisy-Chained Reactor’s Design

**Figure 3:**
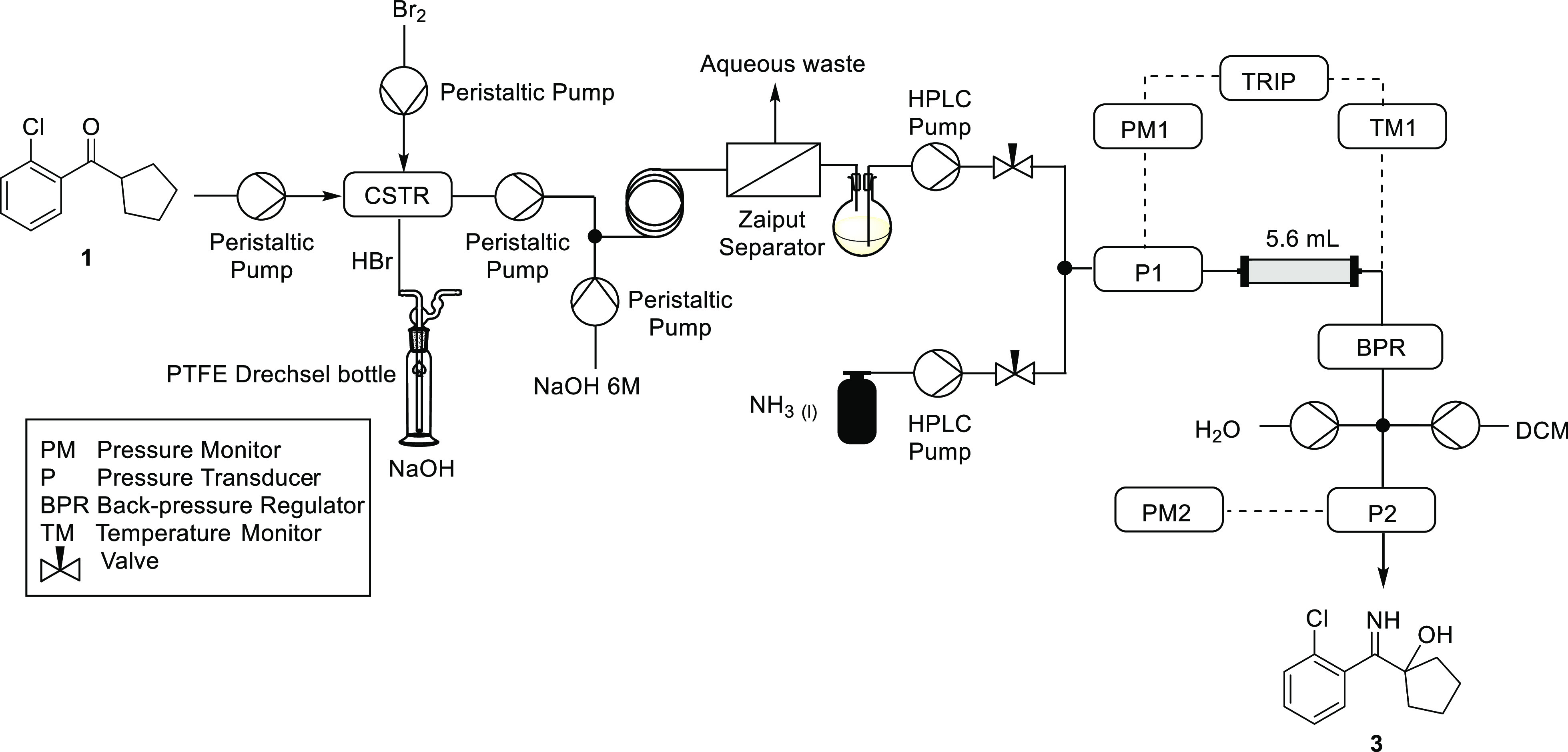
Reactor scheme for the daisy-chained synthesis of α-hydroxy imine **3** from the ketone **1**. The process involved bromination of **1** to form **2**, in-line continuous-flow separation with a liquid–liquid membrane separator, high-pressure amination of the bromide **2** with liquid ammonia, and concomitant rearrangement to form the α-hydroxy imine **3**. Under these conditions, we were able to achieve 90% conversion to the imine **3** over two steps, as determined by ^1^H NMR analysis. Moreover, when the same conditions were repeated without the inclusion of an internal standard, the imine **3** was isolated by precipitation from heptane in 82% yield.

In order to daisy-chain the bromination process into the amination process, it was crucial that all bromine and bromine derivatives were removed from the reaction stream before being passed into the subsequent reactor, which was constructed from stainless-steel piping. This is because bromine can cause stress corrosion cracking of stainless steel. To address this challenge, we adopted an in-line continuous-flow purification, centered on the use of a membrane separator. In order to match the scale of the imination process, the bromination process was scaled down, using a 25 mL reactor volume. A 0.4 M solution of the starting ketone **1** was pumped at 0.05 mL/min and the bromine was made up to 0.44 M in CH_2_Cl_2_ and pumped at the same rate. Under these conditions, the reaction mixture could be pumped out of the reactor at 0.1 mL/min and 0.2 M in substrate (Figure 3).

To the best of our knowledge, there are very few examples where a continuous-flow bromination has been developed that includes an in-line purification before telescoping into a subsequent reactor. In the corresponding batch procedure, an aqueous work-up with Na_2_CO_3_ was employed. In our experience, we found that the resultant crude mixture remained considerably colored following this protocol (an indication of bromine and/or its derivatives) and we were keen to develop a procedure that efficiently removed these contaminants. As such, several aqueous quench solutions were trialed. When sodium thiosulfate was employed, an efficient quench was observed, however, this produced a solid residue, which is undesirable in a flow procedure. Sodium sulfate has been reported to quench bromine effectively, but in our hands we were unable to achieve complete decolorization when this reagent was employed. The optimized procedure was found when sodium hydroxide was utilized. This approach has the benefit of not only neutralizing the hydrogen bromide but also quenching the bromine, to produce bromide and bromite, the latter of which rapidly disproportionates into a second equivalent of bromide and one equivalent of bromate. To assist in the quenching procedure, a 90 cm FEP reactor filled with glass beads was placed before the membrane separator (Figure 3).

### Thermal Rearrangement

**Figure 4:**
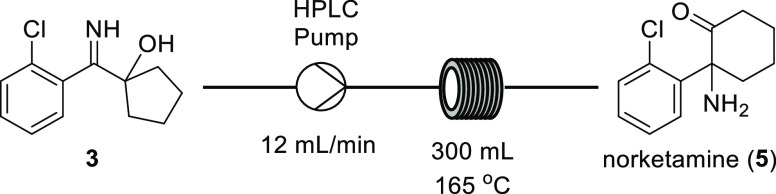
Schematic illustrating the principle of using pressure to superheat an ethanolic solution of α-hydroxy imine **3** to promote the α-iminol rearrangement needed to form norketamine **5** in continuous flow.

Following the imination process, the final step in the synthesis of norketamine (**5**) is an α-iminol rearrangement as shown in Figure 4. We have recently disclosed in a patent a continuous-flow protocol for this transformation.^29^ The principal advantages of this protocol are a reduction in the formation of undesired degradation side products and the simplification of product isolation by use of ethanol superheated in a pressurized system instead of traditionally used high-boiling solvents. Initially, the process was optimized using a FlowSyn reactor, where the starting material solution was administered either directly by a pump or via an injection loop (Table 3).

**Table 3:** Results for the Continuous-Flow α-iminol Rearrangement of the α-hydroxy Imine **3** to Form Norketamine (**5**)

entry	res. time	temp. (°C)	conc. (M)	injection mode	conversion (%)
1	10	200	1.0	pump	64
2	10	200	0.5	pump	95
3	10	200	0.5	injection loop	96
4	10	180	0.5	injection loop	94
5	10	170	0.5	injection loop	94
6	10	160	0.5	injection loop	87
7	20	160	0.5	injection loop	96
8	20	160	0.5	pump	95
9	20	160	1.0	pump	96

Initially, the reaction was attempted at 200 °C (Table 3, Entries 1–3). It was observed that the best conversions were obtained at 0.5 M concentration. It should be noted, however, that at this temperature the precipitation of an insoluble by-product was observed, which was of course undesirable for a continuous-flow procedure. By reducing the temperature, the formation of this by-product was suppressed. At 160 °C, the reaction was completely homogeneous, however, the conversion was reduced to 87%. This was improved by increasing the residence time to 20 min. Under these conditions, the conversion rose to 96 and 95%, respectively, when the starting material solution was administered from the injection loop and the pump, respectively. It was found that at 20 min residence time, the concentration could be increased to 1.0 M, without any loss in yield, hence overcoming the previous reduction in productivity caused by the increased residence time. The optimized procedure involved passing a solution of the imine **3** through a 300 mL Hastelloy tube reactor (CRD Salamander, Cambridge Reactor Design), which was heated to 165 °C. To maximize the concentration of substrate solution and hence the productivity of the process, the imine **3** was employed as a preheated (50 °C) solution in ethanol. At this temperature, 1.4 kg of **3** could be readily dissolved in 2.8 kg of ethanol. The resulting solution was passed through the reactor at a rate of 12 mL/min, which equates to a residence time of 25 min. The output was collected as a single fraction and high-performance liquid chromatography analysis indicated a 95% yield of the target compound norketamine (**5**). The product was not isolated but instead N-Boc protected for use in subsequent steps (see the Supporting Information).

## Conclusions

A three-stage continuous-flow process for the synthesis of norketamine (**5**) is reported. Initially, α-bromination of the key precursor **1** was achieved with quantitative conversion using a CSTR that allowed the safe removal and quenching of the hydrogen bromide by-product. This process was demonstrated on a 0.89 kg/day scale. Next, the subsequent imination process was achieved in excellent yield, representing a rare example of the use of liquid ammonia in a continuous-flow reactor. It was then demonstrated that these processes can be linked via an in-line quench and purification with a liquid–liquid membrane separator. Finally, the last step in the synthesis of norketamine (**5**) was achieved on a 1.4 kg scale via the thermal rearrangement of the α-hydroxy imine **3** in a commercially available tubular flow reactor.

## Supporting Information Available

Description of the materials and methods and analytical data for compounds **2**, **3**, and **4** together with ^1^H and ^13^C NMR spectra for these compounds (PDF)

## References

[ref1] Plutschack M. B.; Pieber B.; Gilmore K.; Seeberger P. H. The Hitchhiker’s Guide to Flow Chemistry. Chem. Rev. 2017, 117, 11796–11893. 10.1021/acs.chemrev.7b00183. 28570059

[ref2] Di Filippo M.; Bracken C.; Baumann M. Continuous Flow Photochemistry for the Preparation of Bioactive Molecules. Molecules 2020, 25, 356 10.3390/molecules25020356. PMC7024297 31952244

[ref3] Newman S. G.; Jensen K. F. The Role of Flow in Green Chemistry and Engineering. Green Chem. 2013, 15, 1456–1472. 10.1039/C3GC40374B.

[ref4] Wiles C.; Watts P. Continuous Flow Reactors: A Perspective. Green Chem. 2012, 14, 38–54. 10.1039/C1GC16022B.

[ref5] Moussavi S.; Chatterji S.; Verdes E.; Tandon A.; Patel V.; Ustun B. Depression, Chronic Diseases, and Decrements in Health: Results from the World Health Surveys. Lancet 2007, 370, 851–858. 10.1016/S0140-6736(07)61415-9. 17826170

[ref6] Henriksson M. M.; Aro H. M.; Marttunen M. J.; Heikkinen M. E.; Isometsä E. T.; Kuoppasalmi K. I.; Lönnqvist J. K. Mental Disorders and Comorbidity in Suicide. Am. J. Psychiatr. 1993, 150, 935–940. 10.1176/ajp.150.6.935. 8494072

[ref7] Nock M. K.; Borges G.; Bromet E. J.; Cha C. B.; Kessler R. C.; Lee S. Suicide and Suicidal Behavior. Epidemiol. Rev. 2008, 30, 133–154. 10.1093/epirev/mxn002. 18653727 PMC2576496

[ref8] Trivedi M. H.; Rush A. J.; Wisniewski S. R.; Nierenberg A. A.; Warden D.; Ritz L.; Norquist G.; Howland R. H.; Lebowitz B.; McGrath P. J.; Shores-Wilson K.; Biggs M. M.; Balasubramani G. K.; Fava M. STAR*D Study Team. Evaluation of Outcomes With Citalopram for Depression Using Measurement-Based Care in STAR*D: Implications for Clinical Practice. Am. J. Psychiatr. 2006, 163, 28–40. 10.1176/appi.ajp.163.1.28. 16390886

[ref9] White P. F.; Trevor J.; Anthony J. T. Ketamine—Its Pharmacology and Therapeutic Uses. J. Am. Soc. Anesthesiol. 1982, 56, 119–136. 10.1097/00000542-198202000-00007. 6892475

[ref10] Zarate C. A. Jr; Singh J. B.; Carlson P. J.; Brutsche N. E.; Ameli R.; Luckenbaugh D. A.; Charney D. S.; Manji H. K. A Randomized Trial of an N-Methyl-D-Aspartate Antagonist in Treatment-Resistant Major Depression. Arch. Gen. Psychiatr. 2006, 63, 856–864. 10.1001/archpsyc.63.8.856. 16894061

[ref11] Zhao X.; Venkata S. L. V.; Moaddel R.; Luckenbaugh D. A.; Brutsche N. E.; Ibrahim L.; Zarate C. A. Jr; Mager D. E.; Wainer I. W. Simultaneous Population Pharmacokinetic Modelling of Ketamine and Three Major Metabolites in Patients with Treatment-Resistant Bipolar Depression. Br. J. Clin. Pharmacol. 2012, 74, 304–314. 10.1111/j.1365-2125.2012.04198.x. 22295895 PMC3630750

[ref12] Zhou W.; Wang N.; Yang C.; Li X.-M.; Zhou Z.-Q.; Yang J.-J. Ketamine-Induced Antidepressant Effects Are Associated with AMPA Receptors-Mediated Upregulation of MTOR and BDNF in Rat Hippocampus and Prefrontal Cortex. Eur. Psychiatr. 2014, 29, 419–423. 10.1016/j.eurpsy.2013.10.005. 24321772

[ref13] Price R. B.; Nock M. K.; Charney D. S.; Mathew S. J. Effects of Intravenous Ketamine on Explicit and Implicit Measures of Suicidality in Treatment-Resistant Depression. Biol. Psychiatr. 2009, 66, 522–526. 10.1016/j.biopsych.2009.04.029. PMC2935847 19545857

[ref14] Price R. B.; Iosifescu D. V.; Murrough J. W.; Chang L. C.; Al Jurdi R. K.; Iqbal S. Z.; Soleimani L.; Charney D. S.; Foulkes A. L.; Mathew S. J. Effects of Ketamine on Explicit and Implicit Suicidal Cognition: A Randomized Controlled Trial in Treatment-Resistant Depression. Depress. Anxiety 2014, 31, 335–343. 10.1002/da.22253. 24668760 PMC4112410

[ref15] DiazGranados N.; Ibrahim L. A.; Brutsche N. E.; Ameli R.; Henter I. D.; Luckenbaugh D. A.; Machado-Vieira R.; Zarate C. A. Rapid Resolution of Suicidal Ideation After a Single Infusion of an N -Methyl- D -Aspartate Antagonist in Patients With Treatment-Resistant Major Depressive Disorder. J. Clin. Psychiatr. 2010, 71, 1605–1611. 10.4088/JCP.09m05327blu. PMC3012738 20673547

[ref16] Ballard E. D.; Ionescu D. F.; Vande Voort J. L.; Niciu M. J.; Richards E. M.; Luckenbaugh D. A.; Brutsché N. E.; Ameli R.; Furey M. L.; Zarate C. A. Improvement in Suicidal Ideation after Ketamine Infusion: Relationship to Reductions in Depression and Anxiety. J. Psychiatr. Res. 2014, 58, 161–166. 10.1016/j.jpsychires.2014.07.027. 25169854 PMC4163501

[ref17] Reinstatler L.; Youssef N. A. Ketamine as a Potential Treatment for Suicidal Ideation: A Systematic Review of the Literature. Drugs R 2015, 15, 37–43. 10.1007/s40268-015-0081-0. PMC4359177 25773961

[ref18] Hashimoto K. Rapid-acting Antidepressant Ketamine, Its Metabolites and Other Candidates: A Historical Overview and Future Perspective. Psychiatr. Clin. Neurosci. 2019, 73, 613–627. 10.1111/pcn.12902. PMC6851782 31215725

[ref19] Stevens C. L.; Elliott R. D.; Winch B. L. Aminoketone Rearrangements. II. The Rearrangement of Phenyl α-Aminoketones. J. Am. Chem. Soc. 1963, 85, 1464–1470. 10.1021/ja00893a018.

[ref20] Stevens C. L.; Thuillier A.; Daniher F. A. Amino Ketone Rearrangements. III. The Rearrangement of α-Hydroxy N-Phenylimines. J. Org. Chem. 1965, 30, 2962–2966. 10.1021/jo01020a018.

[ref21] Stevens C. L.; Klundt I. L.; Munk M. E.; Pillai M. D. Amino Ketone Rearrangements. IV. Thermal Rearrangements of α-Amino Methyl Ketones. J. Org. Chem. 1965, 30, 2967–2972. 10.1021/jo01020a019.

[ref22] Stevens C. L.; Hanson H. T.; Taylor K. G. Amino Ketone Rearrangements. V. A Kinetic Analysis. J. Am. Chem. Soc. 1966, 88, 2769–2774. 10.1021/ja00964a028.

[ref23] Stevens C. L.; Ash A. B.; Thuillier A.; Amin J. H.; Balys A.; Dennis W. E.; Dickerson J. P.; Glinski R. P.; Hanson H. T.; Pillai M. D.; Stoddard J. W. Amino Ketone Rearrangements. VI. Synthesis of 2-Alkylamino-2-Phenylcyclohexanones. J. Org. Chem. 1966, 31, 2593–2601. 10.1021/jo01346a033. 5917446

[ref24] Stevens C. L.; Thuillier A.; Taylor K. G.; Daniher F. A.; Dickerson J. P.; Hanson H. T.; Nielsen N. A.; Tikotkar N. A.; Weier R. M. Amino Ketone Rearrangements. VII. Synthesis of 2-Methylamino-2-Substituted Phenylcyclohexanones. J. Org. Chem. 1966, 31, 2601–2607. 10.1021/jo01346a034.

[ref25] Stevens C. L.; Glenn F. E.; Pillai P. M. Hydroxy Ketone Rearrangements. II. Kinetics and Mechanism of the Thermal Rearrangements of Optically Active .alpha.-Hydroxy Ketones. Example of a Cyclic Three-Component Equilibrium. J. Am. Chem. Soc. 1973, 95, 6301–6308. 10.1021/ja00800a024.

[ref26] Zhang Z.-Q.; Chen T.; Zhang F.-M. Copper-Assisted Direct Nitration of Cyclic Ketones with Ceric Ammonium Nitrate for the Synthesis of Tertiary α-Nitro-α-Substituted Scaffolds. Org. Lett. 2017, 19, 1124–1127. 10.1021/acs.orglett.7b00040. 28206766

[ref27] Kassin V.-E. H.; Gérardy R.; Toupy T.; Collin D.; Salvadeo E.; Toussaint F.; Van Hecke K.; Monbaliu J.-C. M. Expedient Preparation of Active Pharmaceutical Ingredient Ketamine under Sustainable Continuous Flow Conditions. Green Chem. 2019, 21, 2952–2966. 10.1039/c9gc00336c.

[ref28] Becker R.; van den Broek S. A. M. W.; Nieuwland P. J.; Koch K.; Rutjes F. P. J. T. Optimisation and Scale-up of α-Bromination of Acetophenone in a Continuous Flow Microreactor. J. Flow Chem. 2012, 2, 87–91. 10.1556/JFC-D-12-00007.

[ref29] Thomas C. J.; Morris P. J.; Castledine R. A.; Bourne S. Process for Synthesis and Purification of (2R,6R)-Hydroxynorketamine. WO 2019236557 A1, 2019.

