# Supplementary material for: High Yielding Continuous-Flow Synthesis of Norketamine
Source: Org Process Res Dev. 2022 Mar 26;26(4):1145–51. doi: 10.1021/acs.oprd.1c00407 (PMC9098192; doi:10.1021/acs.oprd.1c00407)
Supplement: Supplementary file 1 — op1c00407_si_001.pdf [file op1c00407_si_001.pdf]

High Yielding Continuous-Flow Synthesis of Norketamine

Marcos Veguillas Hernando,[a#] Jonathan C. Moore,[a#] Rowena A. Howie,[b] Richard A. Castledine,[b] Samuel L. Bourne,[b] Gareth N. Jenkins,[b] Peter Licence, [a,c] Martyn Poliakoff,[a] Michael W. George [a]*

[a]School of Chemistry, University of Nottingham, University Park, Nottingham, NG7 2RD. UK

[b]Quotient Sciences, Taylor Drive, Alnwick, Northumberland, NE66 2DH. UK

[c]GSK Carbon Neutral Laboratories for Sustainable Chemistry, University of Nottingham, Nottingham, NG7 2GA. UK

mike.george@nottingham.ac.uk*

Supporting Information

Materials and Methods

All reagents were purchased from a chemical supplier and used without further purification. ^{1}H and ^{13}C NMR spectra were recorded in $CDCl_3$ at ambient temperature using a Bruker AV3400HD (400 MHz), spectrometer. Data are expressed as chemical shifts in parts per million (ppm) relative to residual solvent signals ($CHCl_3$, ^{1}H NMR 7.26), ($CDCl_3$, ^{13}C NMR 77.16) as the internal standard on the δ scale. Infra-red spectra were recorded using a Bruker Tensor 27 FT-IR spectrophotometer using an ATR attachment and their peaks are quoted as υmax in cm^{-1}. HRMS analyses were performed on a Bruker micrOTOFII mass spectrometer (Bruker Daltonik, Bremen, Germany), interfaced to an Agilent 1200 HPLC (Agilent Technologies, Santa Clara, USA). Samples were presented in solution for analysis by Flow Injection, 1 µL of solution being injected into the ion source of the instrument along with a flow of 0.2 mL min^{-1} of 70% methanol/water eluent. The mass spectrometer was operated in electrospray ionisation (ESI) mode at a typical resolving power of 8000. Control of the analysis was performed through Bruker's Compass Open Access QC automated data acquisition and reporting software (v1.3; Bruker Daltonik, Bremen, Germany).

Compound Characterisation

(1-Bromocyclopentyl)(2-chlorophenyl)methanone (2)

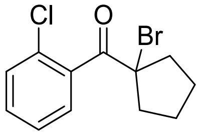

2

^{1}H NMR (400 MHz, CDCl3) δ_H 7.70 (dd, *J* = 7.5, 1.7 Hz, 1H), 7.42 (dd, *J* = 7.9, 1.4 Hz, 1H), 7.36 (app. td, *J* = 7.7, 1.7 Hz, 1H), 7.30 (app. td, *J* = 7.5, 1.4 Hz, 1H), 2.45 – 2.25 (m, 4H), 2.11 – 1.98 (m, 2H), 1.92 – 1.80 (m, 2H); **^{13}C NMR** (101 MHz, $CDCl_3$) δ 199.5, 138.9, 130.8, 130.5, 130.15, 128.3, 126.5, 74.3, 40.4, 23.3; **HRMS** (ESI) m/z calculated for $C_{12}H_{12}BrClNaO$ $[M+Na]^+$ 308.9652, obtained 308.9653.

1-((2-Chlorophenyl)(imino)methyl)cyclopentan-1-ol (3)

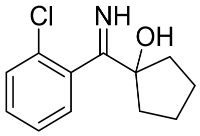

3

m.p. 90.4 – 90.9 °C; **^{1}H NMR** (400 MHz, CDCl3) δ_H 7.42 (dd, *J* = 7.8, 1.5 Hz, 1H), 7.32 (td, *J* = 7.7, 2.0 Hz, 1H), 7.27 (td, *J* = 7.5, 1.6 Hz, 1H), 7.19 (dd, *J* = 7.5, 1.9 Hz, 1H), 2.03 – 1.78 (m, 6H), 1.72 – 1.51 (m, 2H); **^{13}C NMR** (101

MHz, $CDCl_3$) δ 183.5, 138.3, 131.4, 130.1, 130.0, 128.5, 126.4, 85.0, 38.8, 23.7; **HRMS** (ESI) m/z calculated for $C_{12}H_{14}ClNNaO$ $[M+Na]^+$ 246.0656, obtained 246.0654.

tert-Butyl (1-(2-chlorophenyl)-2-oxocyclohexyl)carbamate (S1)

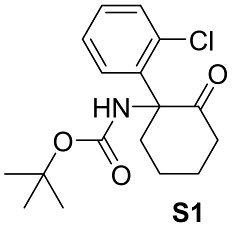

S1

^{1}H NMR (400 MHz, $CDCl_3$) δ_H 7.94 – 7.65 (m, 1H), 7.39 – 7.31 (m, 2H), 7.29 – 7.23 (m, 1H), 6.70 – 6.48 (m, 1H), 3.85 (d, *J* = 14.4 Hz, 1H), 2.46 – 2.24 (m, 2H), 2.14 – 1.97 (m, 1H), 1.89 – 1.58 (m, 5H), 1.31 (s, 9H); **^{13}C NMR** (101 MHz, $CDCl_3$) δ 209.3, 153.6, 135.3, 133.9, 131.7, 131.0, 129.4, 126.3, 79.2, 67.3, 39.6, 38.6, 31.0, 28.4, 22.5; **HRMS** (ESI) m/z calculated for $C_{17}H_{22}ClNNaO_3$ $[M+Na]^+$ 346.1180, obtained 346.1188.

NMR Spectra

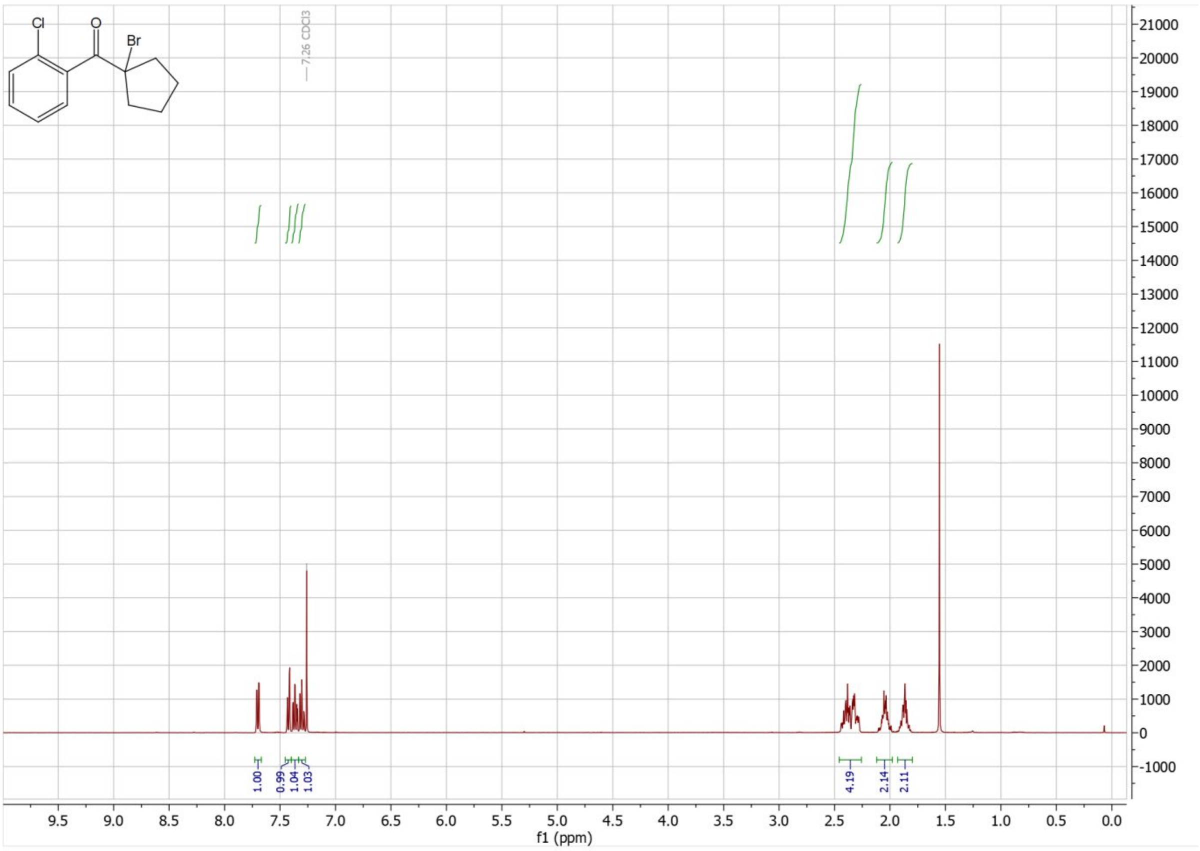

Cl
O
Br
7.26 CDCl3
1.00
0.99
1.04
1.03
4.19
2.14
2.11
f1 (ppm)

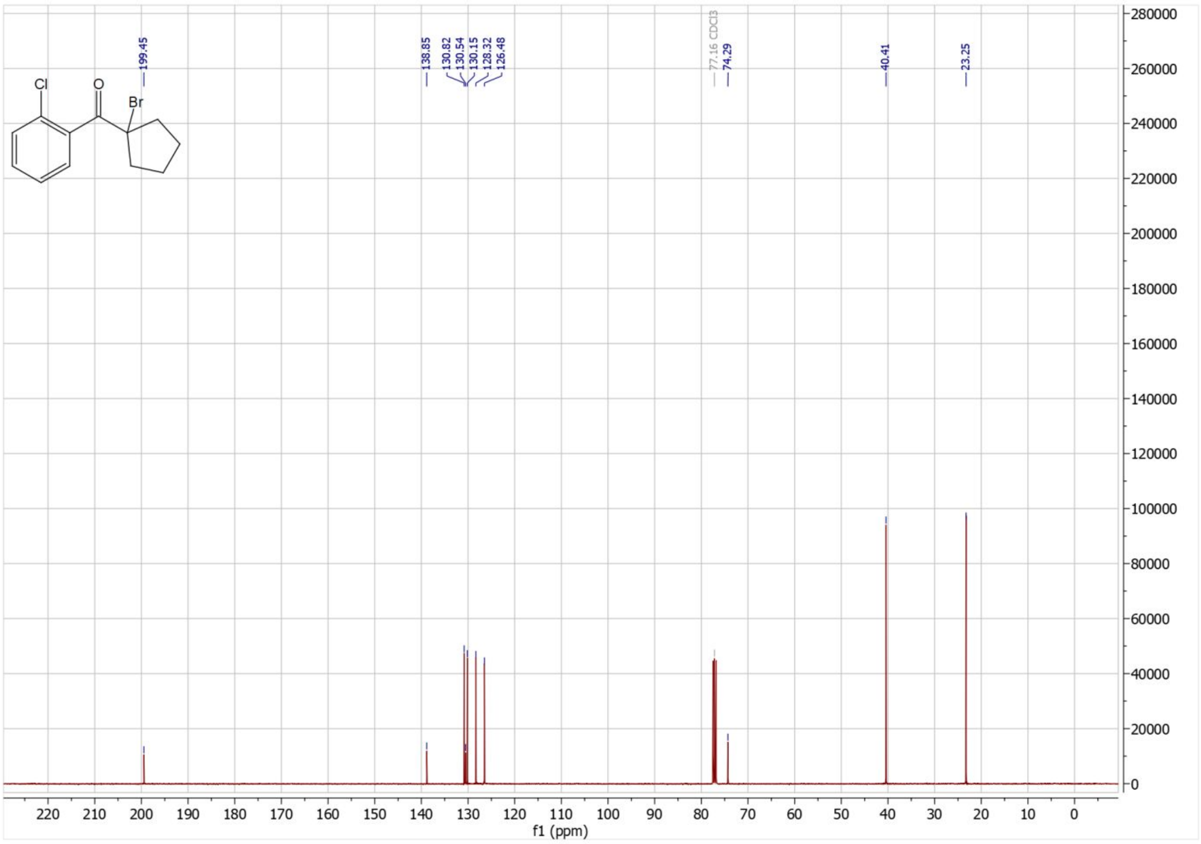

Cl
O
Br
199.45
138.85
130.82
130.54
130.15
128.32
126.48
77.16 CDCl3
74.29
40.41
23.25
f1 (ppm)

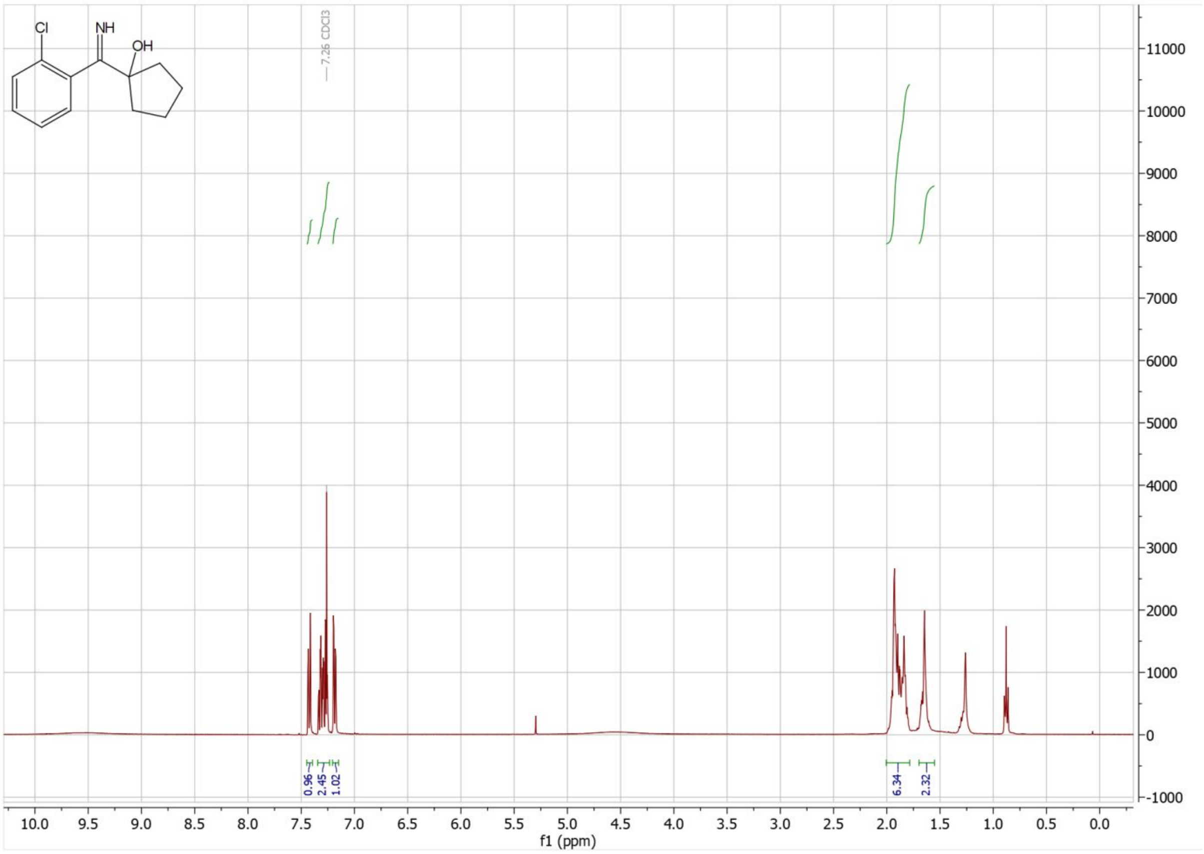
Cl
NH
OH
7.26 CDCl3
0.96
2.45
1.02
6.34
2.32
f1 (ppm)

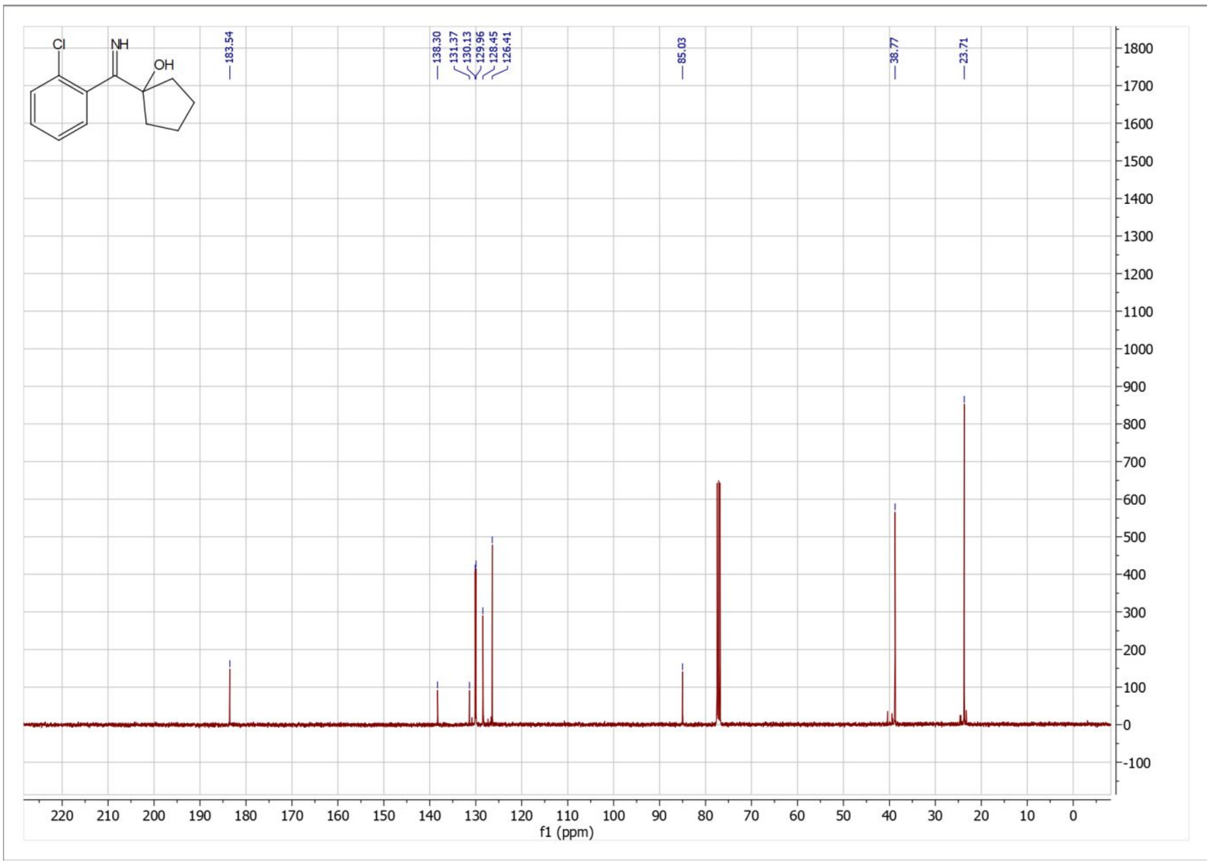
Cl
NH
OH
183.54
138.30
131.37
130.13
129.96
128.45
126.41
85.03
38.77
23.71
f1 (ppm)

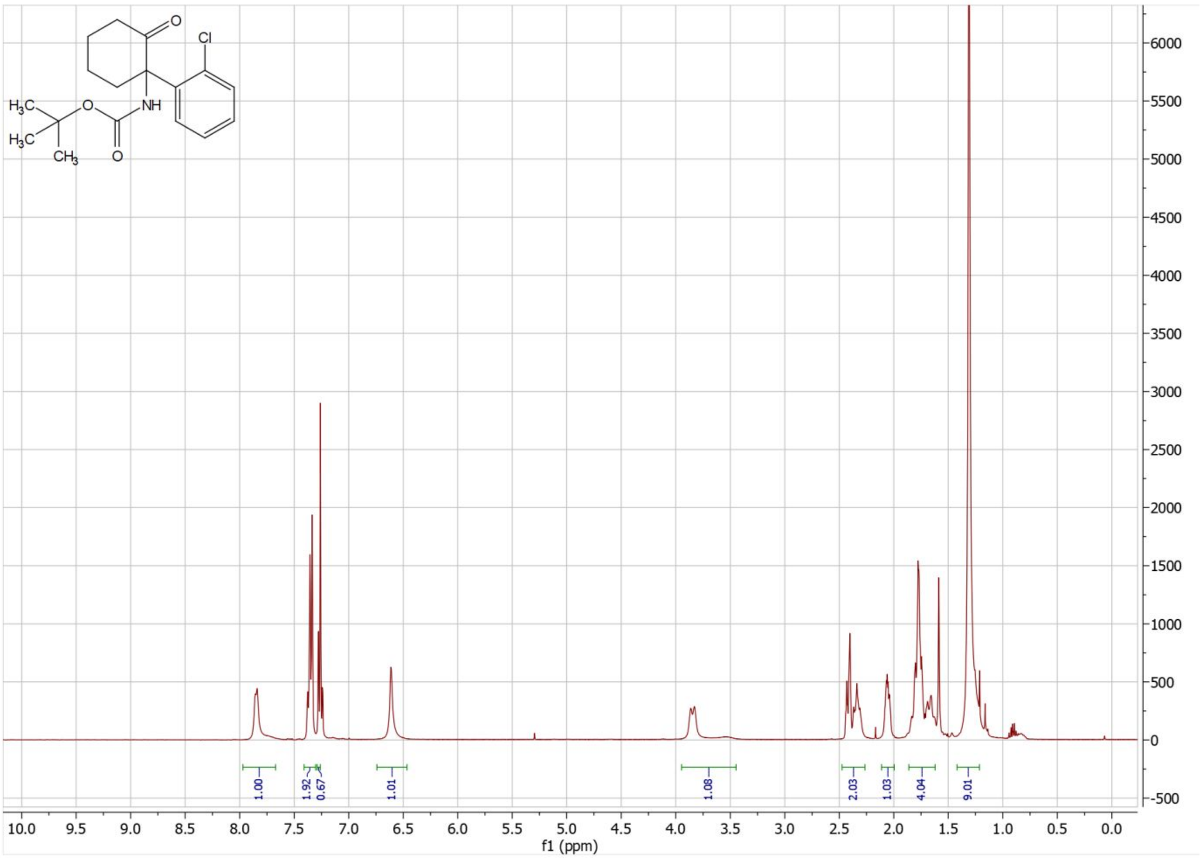
O
Cl
NH
H_3C
H_3C
CH_3
O
O
1.00
1.92
0.67
1.01
1.08
2.03
1.03
4.04
9.01
10.0 9.5 9.0 8.5 8.0 7.5 7.0 6.5 6.0 5.5 5.0 4.5 4.0 3.5 3.0 2.5 2.0 1.5 1.0 0.5 0.0
f1 (ppm)
6000 5500 5000 4500 4000 3500 3000 2500 2000 1500 1000 500 0 -500

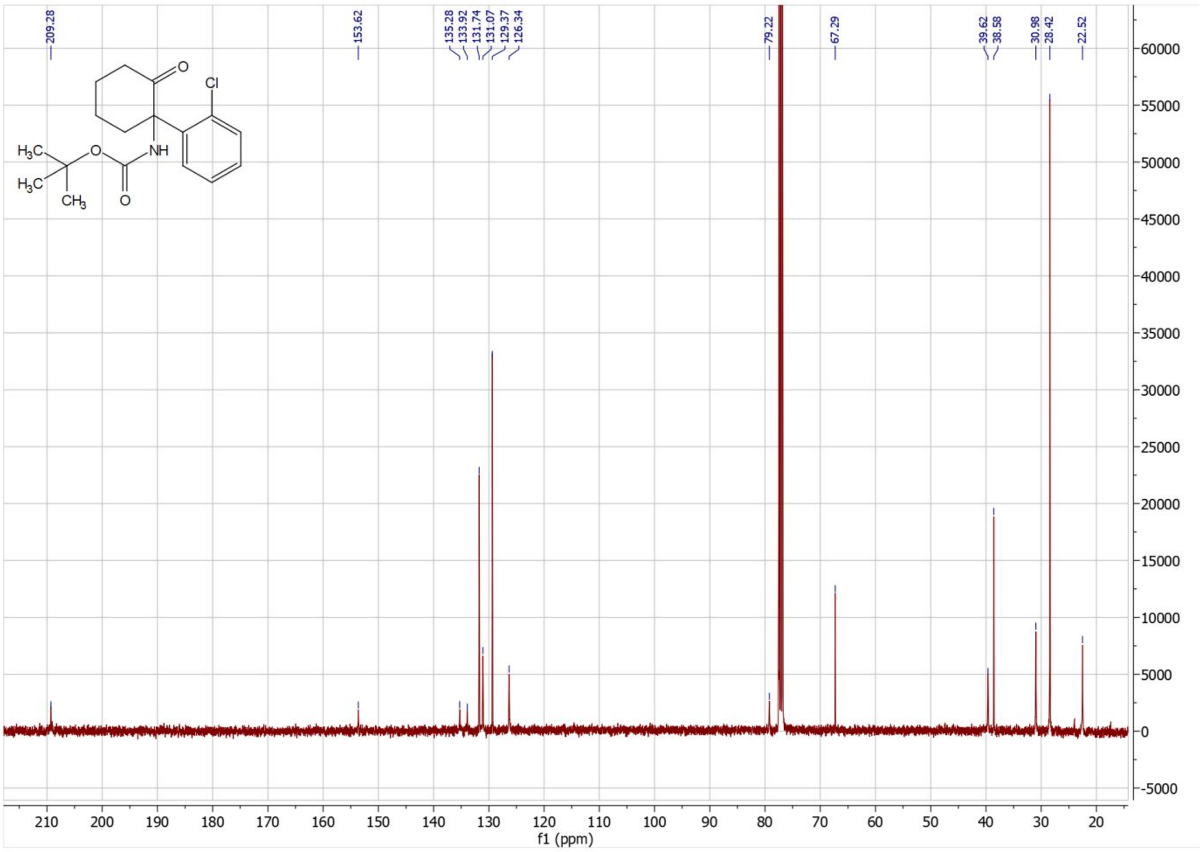
209.28
153.62
135.28
133.92
131.74
131.07
129.37
126.34
79.22
67.29
39.62
38.58
30.98
28.42
22.52
O
Cl
NH
H_3C
H_3C
CH_3
O
O
210 200 190 180 170 160 150 140 130 120 110 100 90 80 70 60 50 40 30 20
f1 (ppm)
60000 55000 50000 45000 40000 35000 30000 25000 20000 15000 10000 5000 0 -5000
